# Phylogenetic Relationships and Structural Conservation of *blaOXA-48*-like Carbapenemase in Multispecies Clinical Strains from an Intensive Care Unit in Pakistan

**DOI:** 10.3390/ijms27125391

**Published:** 2026-06-15

**Authors:** Zeb Hussain, Ambreen Fatima, Asad Karim, Muhammad Jahanzaib, Muhammad Sameer Qureshi, Asma Naim

**Affiliations:** 1 Department of Clinical Laboratory Sciences, Dow Institute of Medical Technology, Dow University of Health Sciences, Karachi 75280, Pakistan; sameer.qureshi@duhs.edu.pk; 2 Department of Microbiology, University of Karachi, Karachi 75270, Pakistan; anaeem@uok.edu.pk; 3 Department of Pathology, Dow International Medical College, Dow University of Health Sciences, Karachi 75280, Pakistan; ambreen.fatima@duhs.edu.pk; 4 Jamil-UR-Rehman Centre for Genome Research, Dr. Panjwani Centre for Molecular Medicine & Drug Research, International Center for Chemical and Biological Sciences, University of Karachi, Karachi 75270, Pakistan; asad.karim@iccs.edu (A.K.); muhammad.jahanzaib@iccs.edu (M.J.)

**Keywords:** antibiotic resistance, *blaOXA-48*, carbapenemase, phylogenetics, structural modeling, horizontal gene transfer, Pakistan strain

## Abstract

The global dissemination of carbapenem resistance is predominantly facilitated by plasmid-mediated carbapenemase genes, notably *blaOXA-48*-like genes. A comprehensive understanding of their evolutionary relationships and structural conservation is essential for monitoring their spread and informing therapeutic strategies. This study aimed to investigate the phylogenetic relationships and structural conservation of *blaOXA-48*-like carbapenemase genes in multiple Gram-negative bacterial species. We analysed *blaOXA-48*-like carbapenemase sequences obtained from a hospital in Pakistan and compared them with globally reported variants retrieved from GenBank. Carbapenemase gene sequences (*blaOXA-48*-like, *blaNDM*, and *blaVIM*) were analyzed using maximum-likelihood phylogenetics (MEGA11, Tamura–Nei model, 1000 bootstrap replicates). Comparative global sequences were retrieved from GenBank. Structural modeling of *blaOXA-48*-like genes was performed using SWISS-MODEL Workspace with the template PDB 3HBR, followed by validation using GMQE, QMEANDisCo, and Ramachandran plot analyses. Phylogenetic analysis revealed a tight clustering of *blaOXA-48*-like genes across *A. baumannii*, *K. pneumoniae*, and *E. meningoseptica*, showing high similarity to globally distributed plasmid-associated sequences. Structural modeling demonstrated strong conservation of the enzyme, with preserved catalytic residues (Ser70, Lys73, Ser118, Trp157, and Tyr211) and minimal structural deviation (RMSD < 0.3 Å). *blaOXA-48*-like carbapenemases exhibit strong phylogenetic conservation and structural stability across species and regions, consistent with the horizontal dissemination of *blaOXA-48*-like genes across bacterial hosts. These findings indicate that *blaOXA-48*-like carbapenemases have high evolutionary stability.

## 1. Introduction

Carbapenem-resistant Gram-negative bacteria constitute a significant threat to global public health, primarily due to the rapid spread of carbapenemase enzymes that degrade last-resort β-lactam antibiotics [1,2]. Among these, class D β-lactamase *blaOXA-48*-like enzymes have emerged as the predominant resistance mechanism, particularly within Enterobacterales and increasingly among non-fermenting Gram-negative pathogens [3,4]. Since its initial identification, the *blaOXA-48*-like gene has demonstrated substantial epidemiological success, largely because of its association with highly transmissible plasmids and its ability to confer resistance while imposing relatively low fitness costs on the bacterial host [5,6]. Unlike other carbapenemases, such as KPC or NDM, *blaOXA-48*-like enzymes exhibit a distinct hydrolytic profile characterized by efficient carbapenem hydrolysis with limited activity against extended-spectrum cephalosporins [7,8]. This biochemical property often results in under-recognition in routine diagnostic settings, thereby facilitating its silent dissemination in clinical settings. The global expansion of *blaOXA-48*-like genes has been extensively documented across Europe, the Middle East, Asia, and Africa, with an increasing prevalence in South Asian healthcare systems, including Pakistan, where the antimicrobial resistance (AMR) burden remains high [9,10]. A key driver of *blaOXA-48*-like dissemination is its frequent localization on mobile genetic elements, particularly conjugative plasmids such as IncL/M-type backbones [11,12]. These plasmids enable horizontal gene transfer (HGT) across diverse bacterial species, including clinically significant pathogens such as *Klebsiella pneumoniae* and *Acinetobacter baumannii* and emerging opportunistic pathogens such as *Elizabethkingia meningoseptica* [13,14]. The capacity for interspecies gene transfer has expanded the ecological niche of *blaOXA-48*-like genes beyond traditional Enterobacterales, reinforcing its role as a globally circulating resistance determinant in humans [15,16].

While epidemiological and molecular surveillance studies have documented the prevalence and dissemination of *blaOXA-48*-like genes, fewer studies have integrated phylogenetic reconstruction with structural analysis to elucidate their evolutionary stability and functional conservation [17,18]. Phylogenetic methodologies offer insights into the relatedness of gene variants across geographic and ecological boundaries, facilitating the identification of transmission pathways and clonal expansion events [19,20]. However, phylogenetic similarity alone does not necessarily indicate functional equivalence, particularly in enzymes, where minor sequence variations can alter substrate specificity or inhibitor susceptibility. In this context, structural biology provides a critical complementary perspective, enabling the evaluation of the three-dimensional protein architecture, active site configuration, and catalytic residue conservation [21,22]. The *blaOXA-48* enzyme belongs to class D β-lactamases and is characterized by a conserved active-site serine residue and a unique carbamylated lysine that is essential for its catalytic activity [21,23]. High-resolution crystallographic studies (e.g., PDB: 3HBR) have elucidated the structural framework of OXA-48, revealing a compact fold and a substrate-binding pocket that accommodates carbapenems. Importantly, the preservation of key catalytic residues, such as Ser70, Lys73, Ser118, Trp157, and Tyr211, is critical for maintaining the enzymatic function [24,25]. Despite the increasing number of reports on *blaOXA-48*-like variants, the extent to which these variants exhibit structural conservation across different bacterial hosts and geographic regions remains insufficiently explored. Understanding whether global dissemination is accompanied by structural divergence or conservation is essential for predicting the long-term effectiveness of β-lactamase inhibitors and guiding drug design strategies [26,27].

Moreover, linking structural stability to phylogenetic clustering can provide deeper insights into the evolutionary pressures that shape resistance gene persistence [28,29]. The clinical relevance of this question is further amplified in high-risk environments, such as intensive care units (ICUs), where antibiotic selection pressure, invasive procedures, and critically ill patient populations create optimal conditions for the emergence and spread of multidrug-resistant (MDR) organisms [30,31]. In such settings, the coexistence of multiple carbapenemase genes, combined with the potential for plasmid exchange, accelerates the evolution of complex resistance phenotypes in bacteria [32]. Therefore, this study aimed to integrate phylogenetic and structural analyses to investigate the evolutionary relationships and molecular conservation of *blaOXA-48*-like carbapenemases identified in multispecies clinical strains. By comparing locally derived sequences with globally reported variants and modeling their three-dimensional structures, we sought to elucidate the patterns of cross-species dissemination, assess the degree of structural conservation within the enzyme, and explore the implications of these findings for AMR surveillance and therapeutic targeting. This combined approach provides a comprehensive framework for understanding the persistence and global success of *blaOXA-48*-like carbapenemases in contemporary clinical settings.

## 2. Results

### 2.1. Phylogenetic Relationships of Carbapenemase Genes

Maximum-likelihood phylogenetic analysis of carbapenemase gene sequences revealed strong clustering of *blaOXA-48*-like variants across multiple bacterial species, including *A. baumannii*, *K. pneumoniae*, and *E. meningoseptica*. The constructed trees demonstrated that the strains in this study were closely related to globally reported sequences retrieved from GenBank, indicating a shared evolutionary lineage.

**Figure 1:**
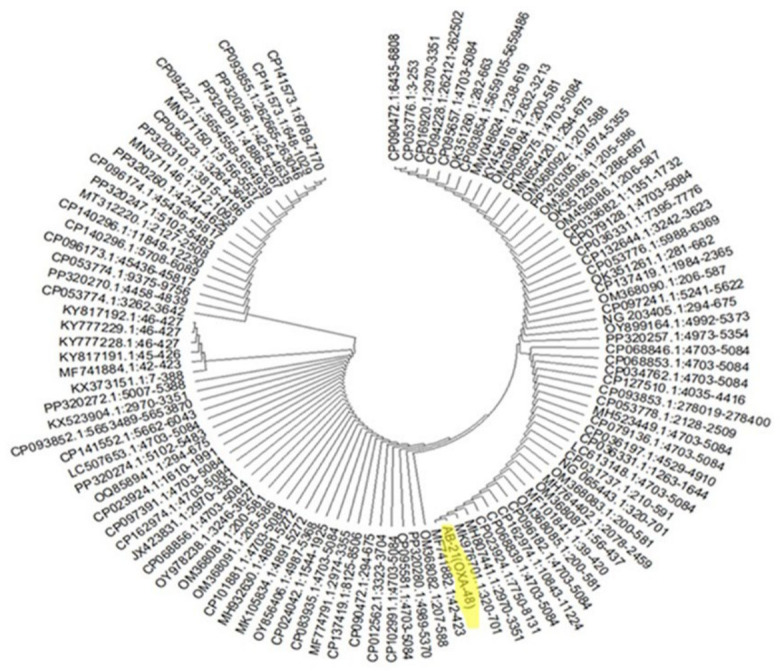
Maximum-likelihood phylogenetic tree (circular dendrogram) of *blaOXA-48* of *A. baumannii* isolate AB-21 (PZ201986) vs. world-wide *blaOXA-48*-producing *A. baumannii* and Enterobacterales. The tree was constructed by means of aligned *blaOXA-48* like sequences with the Tamura–Nei model with 1000 bootstrap replicates. Bootstrap values 70% are displayed on the large branches. Sequences generated in this study are highlighted in yellow.

Notably, *blaOXA-48*-like sequences derived from *A. baumannii* clustered within clades predominantly composed of Enterobacterales-associated plasmid sequences. For example, the sequence from isolate AB-21 showed close phylogenetic proximity to *K. pneumoniae* plasmid sequences reported from Canada and Europe, suggesting probable plasmid-mediated HGT across species boundaries, as shown in Figure 1.

**Figure 2:**
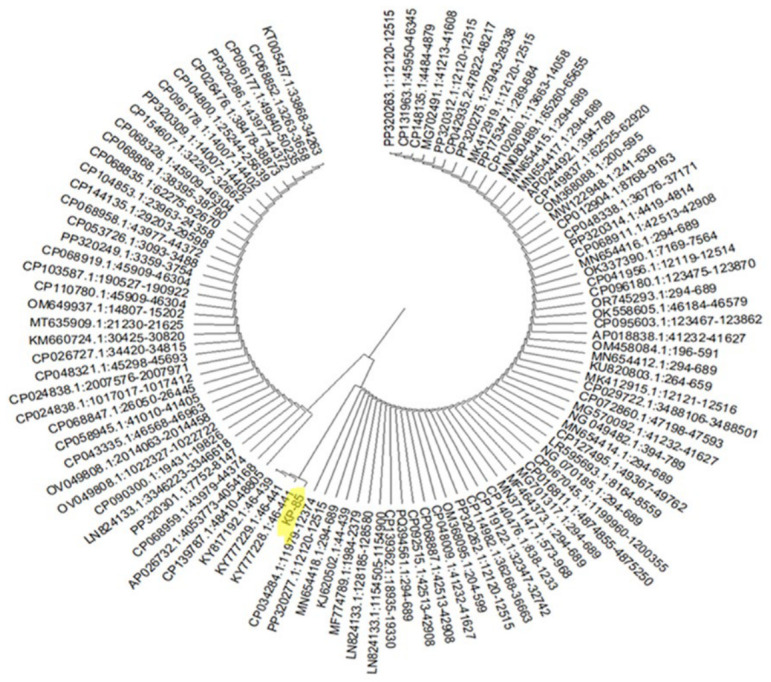
Circular dendrogram of *blaOXA-48*-like sequences from *K. pneumoniae* isolate KP-85 (PZ234370) and global strains, demonstrating clustering with internationally reported plasmid-associated sequences. Sequences generated in this study are highlighted in yellow.

Similarly, *blaOXA-48*-like sequences from *K. pneumoniae* strains were closely grouped with strains originating from the Middle East and South Asia, reflecting regional dissemination with global connectivity as shown in Figure 2. In contrast, sequences from *E. meningoseptica* did not form a distinct lineage but clustered within Enterobacterales-associated clades (Figures S1 and S2), further supporting the interspecies acquisition of resistance genes. Phylogenetic analysis of *blaNDM* genes demonstrated broader clustering across environmental and clinical strains (Figure S3), including those derived from wildlife and environmental reservoirs. This pattern highlights the One Health dimension of AMR, where resistance determinants circulate across clinical, environmental, and animal domains expanded phylogenetic analyses are shown in Supplementary Figures S4–S7.

### 2.2. Global Comparative Analysis and Evidence of Horizontal Gene Transfer

Comparative analysis incorporating global reference sequences revealed that the *blaOXA-48*-like genes identified in this study exhibited high sequence similarity (>99%) with internationally reported plasmid-borne variants. These sequences were from Europe, Asia, and the Middle East, indicating extensive interregional dissemination. A total of 15 carbapenemase gene sequences were identified across multispecies clinical strains, predominantly *blaOXA-48*-like variants, along with *blaNDM*, *blaVIM*, and *blaIMP* genes (Table 1).

The clustering patterns observed in the phylogenetic trees suggest that *blaOXA-48*-like genes are not confined to specific bacterial lineages but are associated with mobile genetic elements that are capable of cross-species transmission. In particular, the presence of nearly identical sequences across taxonomically distinct species is consistent with possible HGT; however, plasmid-mediated transfer requires confirmation by plasmid profiling or whole genome sequencing. Furthermore, the absence of species-specific clustering of *blaOXA-48*-like genes indicates that evolutionary pressures favor gene mobility over host specialization, contributing to the persistence and expansion of this resistance determinant in diverse ecological niches.

### 2.3. Sequence Conservation and Multiple Sequence Alignment

Multiple sequence alignments of *blaOXA-48*-like nucleotide sequences revealed remarkable conservation across all strains, with only minor single-nucleotide polymorphisms (SNPs). Importantly, these variations were confined to nonfunctional regions and did not affect the catalytic domains.

A conserved nucleotide motif corresponding to the enzyme’s active-site region was identified in all sequences, indicating the functional stability of the carbapenemase gene (Figure S8). Multiple sequence alignment of the Sanger-sequenced *blaOXA-48*-like PCR amplicons showed a high degree of conservation across the investigated strains, with only minor nucleotide variations. These partial sequences were used for sequence confirmation and fragment-based phylogenetic comparison rather than for full-length protein translation. Therefore, no inference regarding the full-length amino acid size was made from the ~438 bp amplicons.

The conserved nucleotide patterns observed within the amplified region support the identity and high sequence conservation of *blaOXA-48*-like genes among the studied strains. No major disruptive changes were observed within the sequenced fragment.

At the amino acid level, all key catalytic residues were strictly conserved across strains, including: Ser70, Lys73, Ser118, Trp157, and Tyr211. No truncations, frameshifts, or deleterious mutations were detected, confirming the integrity of the encoded enzymes.

### 2.4. Conservation Analysis of OXA β-Lactamase Genes

Multiple sequence alignment (MSA) of the OXA 48 gene sequences of OXA-48 β-lactamase bacterial strains in BioEdit software was performed using Clustal W version. Compatibility showed some commonality, as well as disagreement of particular nucleotides and amino acids that were specific to each isolate. All sequences shared common nucleotide patterns, particularly in the 5′ and middle coding regions. For example, in all sequences, there was a stretch containing the GATATCGCCGCTTGG and nearly no variation. The repeated CGT, GCG, and TGG were also observed in all sequences. The findings indicate that even though there are sequence divergences, OXA β-lactamase genes have retained some areas by evolution to maintain some functional roles, as shown in Figure S8.

### 2.5. Structural Modeling of blaOXA-48-like Enzyme

**Figure 3:**
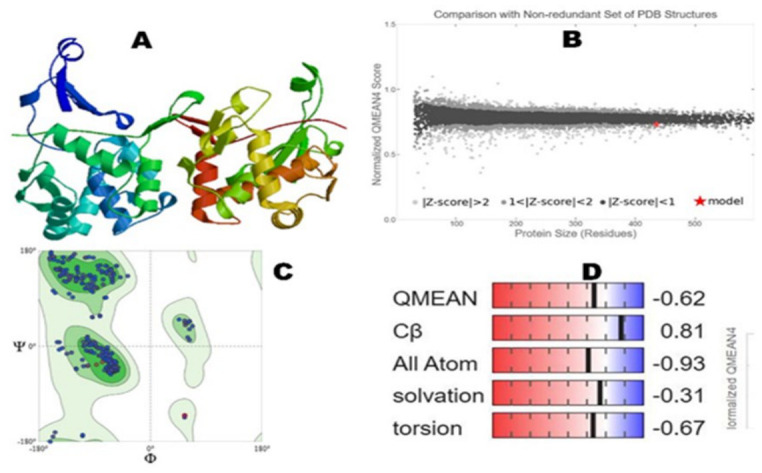
Quality assessment and structural modeling of the predicted OXA-48-like protein model. (**A**) Three-dimensional ribbon representation of the predicted protein model, colored from the N-terminus to the C-terminus. (**B**) QMEAN comparison plot showing the position of the predicted model in relation to a non-redundant set of experimentally solved protein structures deposited in the Protein Data Bank. (**C**) Ramachandran plot showing the distribution of backbone dihedral angles, with most residues located in favored or allowed regions, indicating acceptable stereochemical quality. (**D**) QMEAN component-wise quality score plot showing the global and component-level quality parameters of the predicted model.

Homology modeling of the *blaOXA-48*-like enzyme using the SWISS-MODEL platform demonstrated high structural similarity to the reference crystal structure (PDB: 3HBR). The generated model exhibited a GMQE score of approximately 0.85, indicating strong model reliability. Superimposition of the modeled structure with the template revealed minimal deviation, with a root mean square deviation (RMSD) of less than 0.3 Å, confirming near-identical three-dimensional conformation (Figure 3). The enzyme retained its characteristic class D β-lactamase fold, including the conserved active-site architecture essential for catalytic activity.

### 2.6. Structural Validation and Active-Site Conservation

Structural validation using Ramachandran plot analysis showed that more than 90% of the residues were located in the favored regions, with no residues in the disallowed regions, confirming the excellent stereochemical quality of the model (Figure 3). A detailed inspection of the active site demonstrated the complete conservation of catalytic residues, including nucleophilic Ser70 and carbamylated Lys73, which are critical for β-lactam hydrolysis. The spatial orientations of these residues remained unchanged compared to those of the reference structure.

These findings indicate that structural conservation is maintained despite phylogenetic diversity, suggesting that the functional activity of *blaOXA-48*-like enzymes remains stable across different bacterial hosts.

### 2.7. Functional Implications of Structural Conservation

The absence of structural variation in the active site suggests that the *blaOXA-48*-like enzymes retain consistent catalytic efficiency and substrate-binding characteristics across strains. This structural stability likely contributes to the persistence and global success of this resistance determinant in the Enterobacteriaceae. The conserved active-site architecture indicates that resistance is primarily maintained through dissemination of the *blaOXA-48*-like gene, differences in expression levels, and co-occurrence with additional resistance determinants, rather than through major structural changes in the enzyme. These findings indicate that resistance is primarily driven by gene dissemination, differential expression levels, and co-occurrence with other resistance determinants.

## 3. Discussion

This study offers a comprehensive phylogenetic and structural examination of *blaOXA-48*-like carbapenemases found in clinical strains from multiple species, emphasizing their evolutionary stability and widespread dissemination in clinical settings. Phylogenetic analysis revealed that the *blaOXA-48*-like sequences identified in this study were closely related to globally reported variants, reinforcing the idea that these resistance genes belong to a highly conserved and widely distributed gene pool [11,33]. A significant finding was the lack of species-specific grouping in the phylogenetic trees, where *blaOXA-48*-like sequences from *A. baumannii*, *K. pneumoniae*, and *E. meningoseptica* were mixed with sequences from various geographic locations. This pattern strongly indicates that HGT is the main mechanism behind the spread of *blaOXA-48*-like genes, rather than clonal expansion within a single bacterial lineage [34,35]. The association of *blaOXA-48*-like genes with plasmid-mediated mobility further underscores their ability to rapidly spread between species, especially in high-risk environments such as intensive care units (ICUs) [36]. The phylogenetic clustering of *blaNDM* sequences with strains from the environment and wildlife further highlights the One Health aspect of AMR, emphasizing the interconnectedness of clinical, environmental, and animal reservoirs of resistance-associated genes [37,38]. These findings suggest that resistance genes circulating in hospital settings may originate from or be continuously exchanged with other ecological niches. At the structural level, homology modeling showed a high degree of conservation in the *blaOXA-48*-like enzyme, with minimal deviation from the reference crystal structure (PDB ID: 3HBR). The preservation of key catalytic residues, including Ser70, Lys73, Ser118, Trp157, and Tyr211, indicates that the enzymatic function of *blaOXA-48*-like remains stable across different bacterial strains and host species. The low RMSD values and favorable Ramachandran plot distribution further confirmed the structural integrity and reliability of the modeled protein. The combined phylogenetic and structural findings suggest that the success of *blaOXA-48*-like carbapenemases is not driven by structural diversification but by genetic mobility and efficient dissemination mechanisms. Unlike other resistance determinants that evolve through frequent mutations affecting substrate specificity, *blaOXA-48*-like enzymes appear to maintain a conserved functional core while spreading across diverse bacterial taxa. This evolutionary stability has important implications for managing AMR. The conserved active-site architecture suggests that *blaOXA-48*-like enzymes remain predictable molecular targets for inhibitor design, although their widespread distribution and frequent co-occurrence with other carbapenemases (e.g., *blaNDM*) may limit their therapeutic effectiveness in clinical settings. Furthermore, the detection of *blaOXA-48*-like genes in non-traditional hosts, such as *E. meningoseptica*, expands the known host range of this resistance determinant, indicating that opportunistic pathogens may serve as additional reservoirs of carbapenemase genes. This highlights the importance of continuous molecular surveillance to monitor emerging transmission pathways. Overall, this study underscores that the persistence and global success of *blaOXA-48*-like carbapenemases are primarily attributable to their structural conservation and horizontal dissemination, rather than adaptive structural evolution. The integration of phylogenetic and structural approaches provides a comprehensive framework for understanding the dynamics of AMR at the genetic and molecular levels.

One limitation of the present study is that 16S rRNA gene sequencing was not performed for species-level confirmation. However, species identification was strengthened by subculture of stored strains followed by MALDI-TOF MS prime confirmation, in addition to the initial biochemical and API-based identification. Future studies using 16S rRNA gene sequencing or whole-genome sequencing would provide further genotypic confirmation and allow detailed strain-level comparison and characterization of mobile genetic elements.

## 4. Materials and Methods

### 4.1. Study Design, Source of Strains, and Species Identification

The present study included carbapenem-resistant Gram-negative clinical strains recovered from patients with ventilator-associated pneumonia (VAP) at a tertiary-care hospital in Karachi, Pakistan, between July 2022 and January 2023. These isolates were obtained from clinical respiratory specimens and selected for molecular confirmation, phylogenetic comparison, and structural analysis of carbapenemase genes.

A total of 20 representative carbapenem-resistant strains were included in the final analysis, comprising 11 *A. baumannii*, 7 *K. pneumoniae*, and 2 *E. meningoseptica* strains. The strains were selected based on the carbapenem resistance profile, species representation, and availability of stored cultures for further molecular analysis.

Initial identification was performed using standard microbiological procedures, including colony morphology, Gram staining, and routine biochemical testing, followed by API 20E and API 20NE systems, according to the manufacturer’s instructions. To strengthen species-level identification, stored cultures were subcultured to obtain pure colonies, which were subsequently re-confirmed using matrix-assisted laser desorption/ionization time-of-flight mass spectrometry (MALDI-TOF MS). Only strains with reliable species-level identifications were included in the final analysis.

Publicly available GenBank sequences were not used as the study specimens. These sequences were used only as comparative reference sequences for phylogenetic analysis. An overview of the methodological workflow is presented in Supplementary Figure S9.

### 4.2. Antimicrobial Susceptibility Testing

Antimicrobial susceptibility testing (AST) was performed as part of the phenotypic characterization of the selected Gram-negative clinical strains. Susceptibility to β-lactams, carbapenems, aminoglycosides, fluoroquinolones, and other clinically relevant antimicrobial agents was assessed using standard laboratory procedures according to Clinical and Laboratory Standards Institute (CLSI) guidelines. Minimum inhibitory concentrations (MICs) for carbapenem antibiotics were determined where applicable, and results were interpreted using current CLSI breakpoints.

Strains showing non-susceptibility to at least one agent in three or more antimicrobial categories were classified as multidrug-resistant (MDR). Carbapenem-resistant strains were selected for further molecular screening of carbapenemase genes, including *blaOXA-48*-like, *blaNDM*, *blaVIM*, *blaIMP*, and *blaKPC*. The AST profile was used only for phenotypic characterization and selection of representative strains for downstream molecular, phylogenetic, and structural analyses [16].

### 4.3. PCR Detection of Carbapenemase Genes

All 20 strains were screened for the *blaOXA-48*-like, *blaNDM*, *blaVIM*, *blaIMP*, and *blaKPC* carbapenemase genes. Genomic DNA was extracted using a commercial kit (Qiagen, Hilden, Germany) according to the manufacturer’s protocol. PCRs were performed in 25 µL reactions with 50 ng template DNA, 0.2 µM primers, 200 µM dNTPs, 2.5 µL 10× buffer, 1.5 mM MgCl_2_, and 1 U Taq polymerase (Thermo Fisher Scientific, Vilnius, Lithuania). Cycling included denaturation at 94 °C for 4 min; 35 cycles of 94 °C for 60 s, annealing for 40 s, 72 °C for 50 s; and a final 72 °C for 5 min. The primer details are presented in Supplementary Table S1 [39]. Amplicons were analyzed on 1.5% agarose gels with Gel Red and a 100 bp DNA ladder. Negative and positive controls were included in each experiment.

### 4.4. Sanger Sequencing and Sequencing Confirmation

PCR-positive amplicons of carbapenemase genes were purified using the Gene JET PCR Purification Kit (Thermo Fisher Scientific, Vilnius, Lithuania)according to the manufacturer’s instructions. Purified PCR products were subjected to Sanger sequencing using the same forward and reverse primers used for PCR amplification.

The obtained forward and reverse sequence reads were checked for chromatogram quality, trimmed to remove low-quality regions, and assembled to generate consensus sequences. The resulting sequences were compared with reference sequences available in the NCBI GenBank database using NCBI BLASTn web server (National Center for Biotechnology Information, Bethesda, MD, USA) to confirm gene identity. Sequences showing high similarity with known carbapenemase genes were considered confirmed.

The confirmed partial carbapenemase gene sequences were used for downstream multiple sequence alignment and phylogenetic analysis. The sequence lengths reported in Table 1 represent PCR-amplified partial gene fragments and not full-length coding sequences.

### 4.5. Phylogenetic Analysis of Carbapenemase Genes

We assessed the global relatedness of carbapenemase genes from our strains by analyzing their PCR-amplified sequences. Representative *blaVIM*, *blaOXA-48*-like, and *blaNDM* amplicons from our strains were Sanger sequenced in both directions, then aligned to GenBank reference sequences. Alignments were performed using Clustal Omega v1.2.4 (EMBL-EBI), and maximum-likelihood phylogenetic trees were built with MEGA version 11.0.13 using the Tamura–Nei model. Branch support was measured using 1000 bootstrap replicates, and nodes with values ≥ 70% were considered robust. The trees include GenBank accession numbers, source organisms, and countries for references. This allowed for a comparison of the genes of our ICU strains with those of global sources. Figure 1 and Figure 2 (see Section 2) show representative trees for *OXA-48*-like genes. Additional trees are shown in the Supplementary Materials (Figures S1–S7). We interpreted the clustering patterns to infer clonality and possible HGT.

### 4.6. Homology Modeling of OXA-48

To evaluate the structural conservation of the *blaOXA-48*-like enzyme, we constructed a homology model based on a *blaOXA-48*-like reference crystal structure. A *blaOXA-48*-like gene from one of our strains was used (amino acid sequence derived from PCR product sequencing). The sequence was submitted to SWISS-MODEL Workspace (SIB Swiss Institute of Bioinformatics, Basel, Switzerland), with the *blaOXA-48*-like crystal structure (PDB 3HBR, 1.9 Å resolution) as the template. Model quality was assessed using SWISS-MODEL’s GMQE and QMEANDisCo global scores and the local distance difference test (lDDT) for structural accuracy. The model was visualized in PyMOL v2.5 (Schrödinger LLC, New York, NY, USA)and superimposed on the 3HBR template to examine rmsd and active-site alignment. Key catalytic residues (notably the class D β-lactamase motif Ser70–Lys73 and other active-site residues) were examined for positional conservation. A Ramachandran plot was generated (using PROCHECK v3.5.4to evaluate the backbone dihedral angles, and the proportion of residues in the favored versus disallowed regions was recorded as a measure of stereochemical quality. In addition, component-wise quality metrics (per-residue error estimates) were reviewed. Figure 3 (Section 2) shows the homology model and validation analyses.

## 5. Conclusions

This study demonstrates that *blaOXA-48*-like carbapenemases exhibit strong phylogenetic clustering and high structural conservation across multiple clinical species. The close relationship between these sequences and globally reported variants confirms their widespread dissemination via HGT, particularly through plasmid-mediated mechanisms.

Structural analysis revealed the preservation of the catalytic architecture, indicating that these enzymes maintain functional stability, despite their broad distribution across diverse bacterial hosts. This suggests that the persistence of *blaOXA-48*-like carbapenemases is primarily driven by gene mobility rather than structural adaptations.

The integration of phylogenetic and structural analyses highlights the importance of molecular surveillance in understanding the dynamics of AMR. These findings provide valuable insights into the evolutionary behavior of *blaOXA-48*-like enzymes and support their continued relevance as targets for therapeutic interventions and the development of inhibitors.

## Figures and Tables

**Table 1:** Characteristics of carbapenemase gene sequences identified in multispecies clinical strains.

S. No.	Isolate ID	Bacterial Species	Gene Detected	PCR Amplicon Length (bp)	GenBank Accession No.	Status
1	KP-04	*K. pneumoniae*	*blaOXA-48*-like	~438	PZ224593	Confirmed
2	KP-92	*K. pneumoniae*	*blaOXA-48*-like	~438	PZ234371	Confirmed
3	KP-85	*K. pneumoniae*	*blaOXA-48*-like	~438	PZ234370	Confirmed
4	KP-81	*K. pneumoniae*	*blaOXA-48*-like	~438	PZ234369	Confirmed
5	EK-83	*E. meningoseptica*	*blaOXA-48*-like	~438	PZ234368	Confirmed
6	EK-61	*E. meningoseptica*	*blaOXA-48*-like	~438	Not yet assigned	Accession number pending
7	AB-56	*A. baumannii*	*blaOXA-48*-like	~438	PZ228821	Confirmed
8	AB-46	*A. baumannii*	*blaOXA-48*-like	~438	PZ224592	Confirmed
9	AB-43	*A. baumannii*	*blaOXA-48*-like	~438	PZ204738	Confirmed
10	AB-21	*A. baumannii*	*blaOXA-48*-like	~438	PZ201986	Confirmed
11	AB-57	*A. baumannii*	*blaNDM*	~621	PZ204737	Confirmed
12	AB-04	*A. baumannii*	*blaOXA-48*-like	~438	PZ023851	Confirmed
13	AB-04	*A. baumannii*	*blaVIM*	~390	Not yet assigned	Accession number pending
14	AB-40	*A. baumannii*	*blaOXA-48*-like	~438	Not yet assigned	Accession number pending
15	AB-40	*A. baumannii*	*blaIMP*	~232	Not yet assigned	Accession number pending

Note: Some strains harbored multiple carbapenemase genes and are therefore listed more than once. The listed sequence lengths represent Sanger-sequenced PCR amplicons/partial gene fragments used for molecular confirmation and phylogenetic comparison. They do not represent full-length carbapenemase coding sequences.

## Data Availability

The original contributions presented in this study are included in the article/Supplementary Materials. Further inquiries can be directed to the corresponding author.

## Supplementary Materials

The following supporting information can be downloaded at: https://www.mdpi.com/article/10.3390/ijms27125391/s1. Reference [39] is cited in the supplementary materials.
