# Supplementary material for: Phylogenetic Relationships and Structural Conservation of blaOXA-48-like Carbapenemase in Multispecies Clinical Strains from an Intensive Care Unit in Pakistan
Source: Int J Mol Sci. 2026 Jun 15;27(12):5391. doi: 10.3390/ijms27125391 (PMC13299608; doi:10.3390/ijms27125391)
Supplement: Supplementary file 1 [file ijms-27-05391-s001.zip › ijms-4333470-supplementary.pdf]

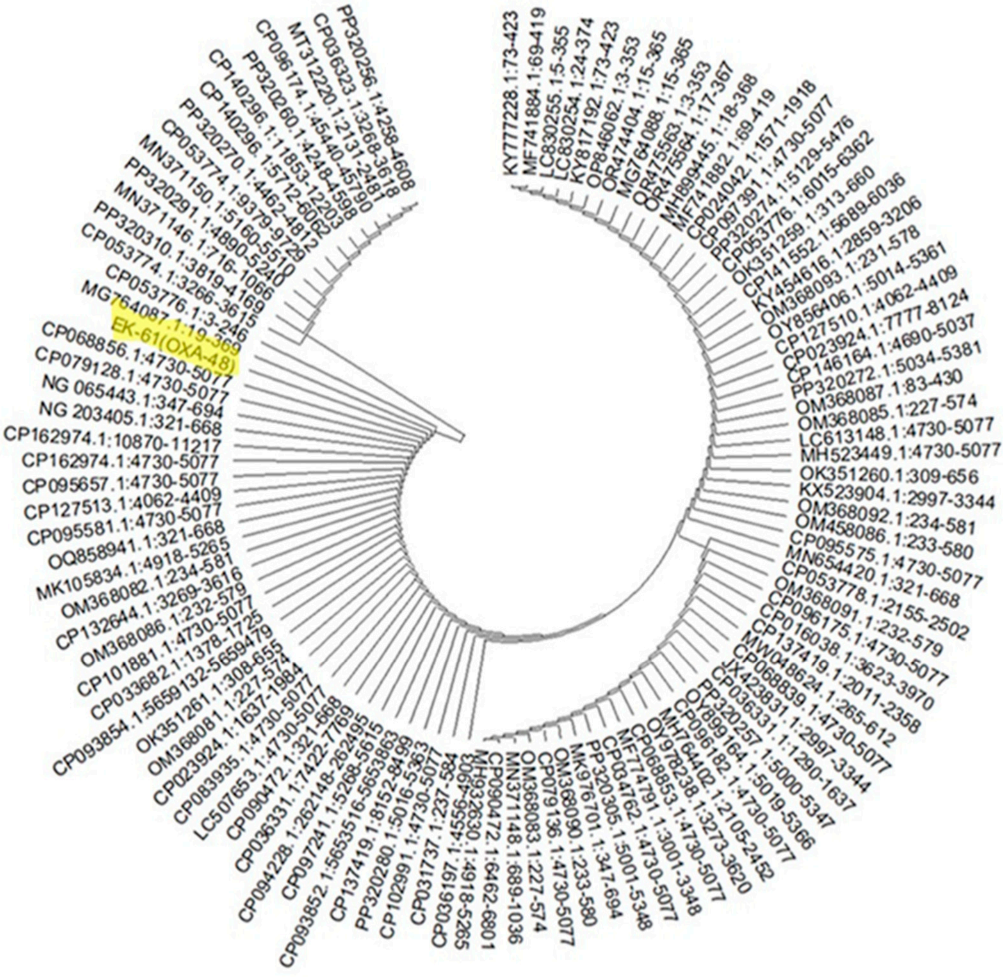

Figure S1. Maximum-likelihood phylogenetic tree showing the relationship between *Elizabethkingia meningoseptica* isolate EK-61 (PZ234368) and global *blaOXA-48*-like and *blaNDM*-producing strains. The tree was constructed using the Tamura–Nei model with 1,000 bootstrap replicates. Sequence-es generated in this study are highlighted in yellow

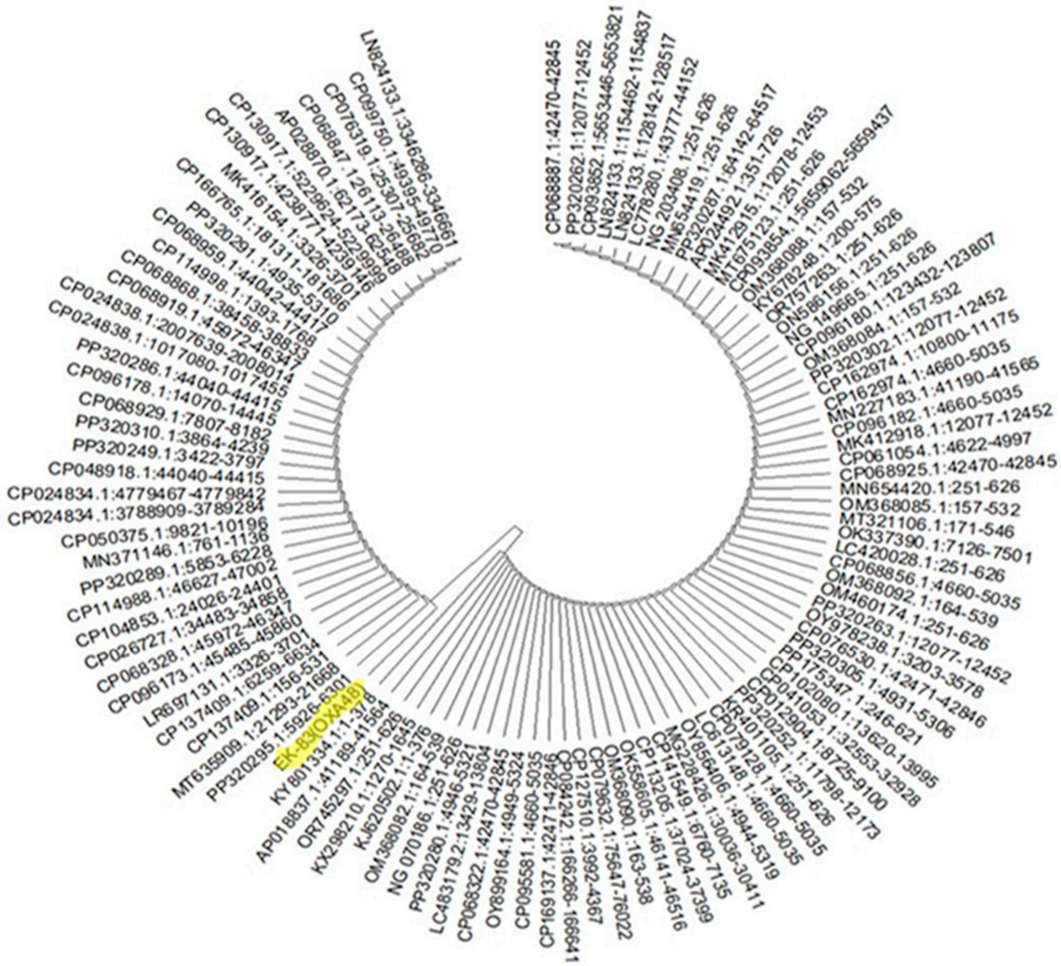

Figure S2. Maximum-likelihood phylogenetic tree showing the relationship between Elizabethkingia meningoseptica isolate EK-83 (PZ234368) and global *blaOXA-48*-like and blaNDM-producing strains. The tree was constructed using the Tamura–Nei model with 1,000 bootstrap replicates. Sequence-es generated in this study are highlighted in yellow

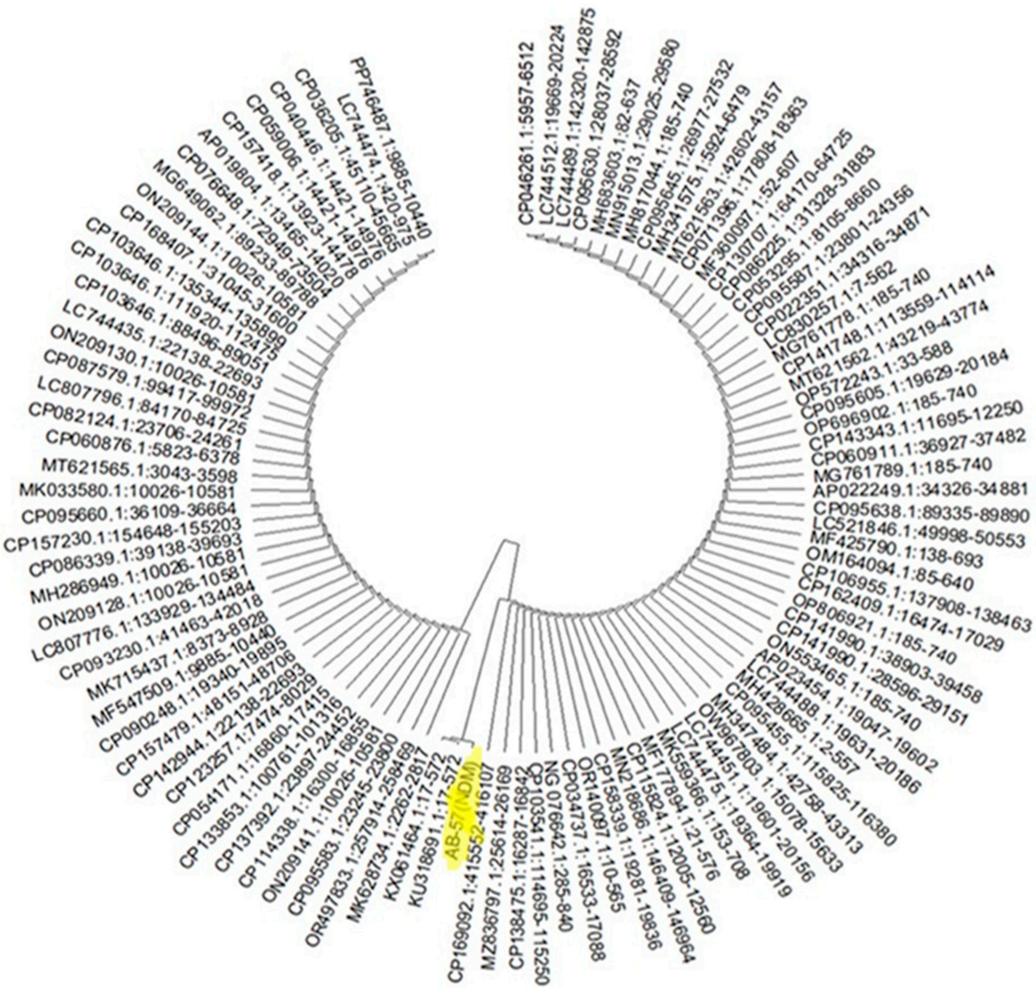

Figure S3. Maximum-likelihood phylogenetic tree of *blaNDM*-1 sequences from *Acinetobacter baumannii* isolate AB-57 (PZ204737) and global reference strains. The tree was constructed using the Tamura–Nei model with 1,000 bootstrap replicates. Sequences generated in this study are highlighted in yellow

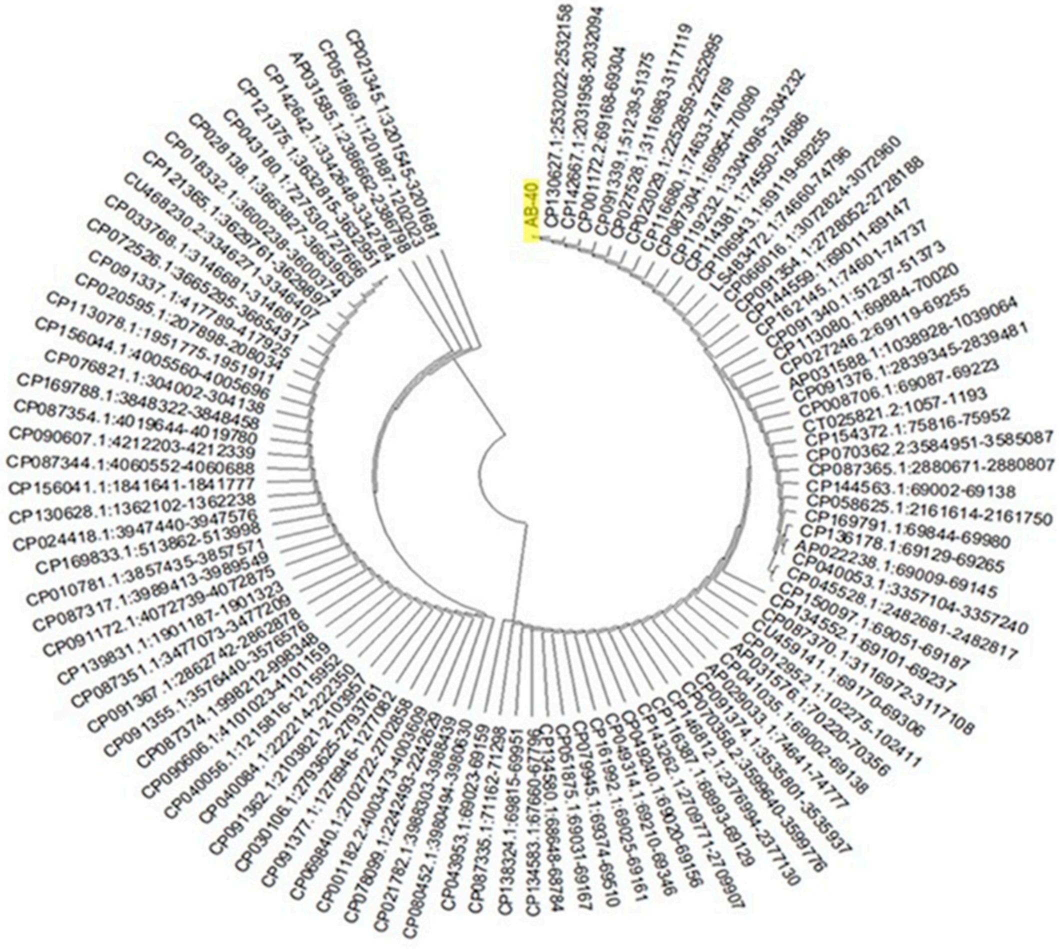

Figure S4. Phylogenetic tree (circular dendrogram) of the maximum-likelihood using *blaOXA*-48 like of isolates of *A. baumannii* of *A. baumannii* worldwide based on *blaOXA-48* like sequences. Close clustering of AB-40 with European and East Asian strains is witnessed in the tree showing a conserved *blaOXA-48* like lineage. It was done with the Tamura-Nei model and with 1,000 bootstrap replicas; the values of bootstrap 70% are shown. Sequences generated in this study are highlighted in yellow.

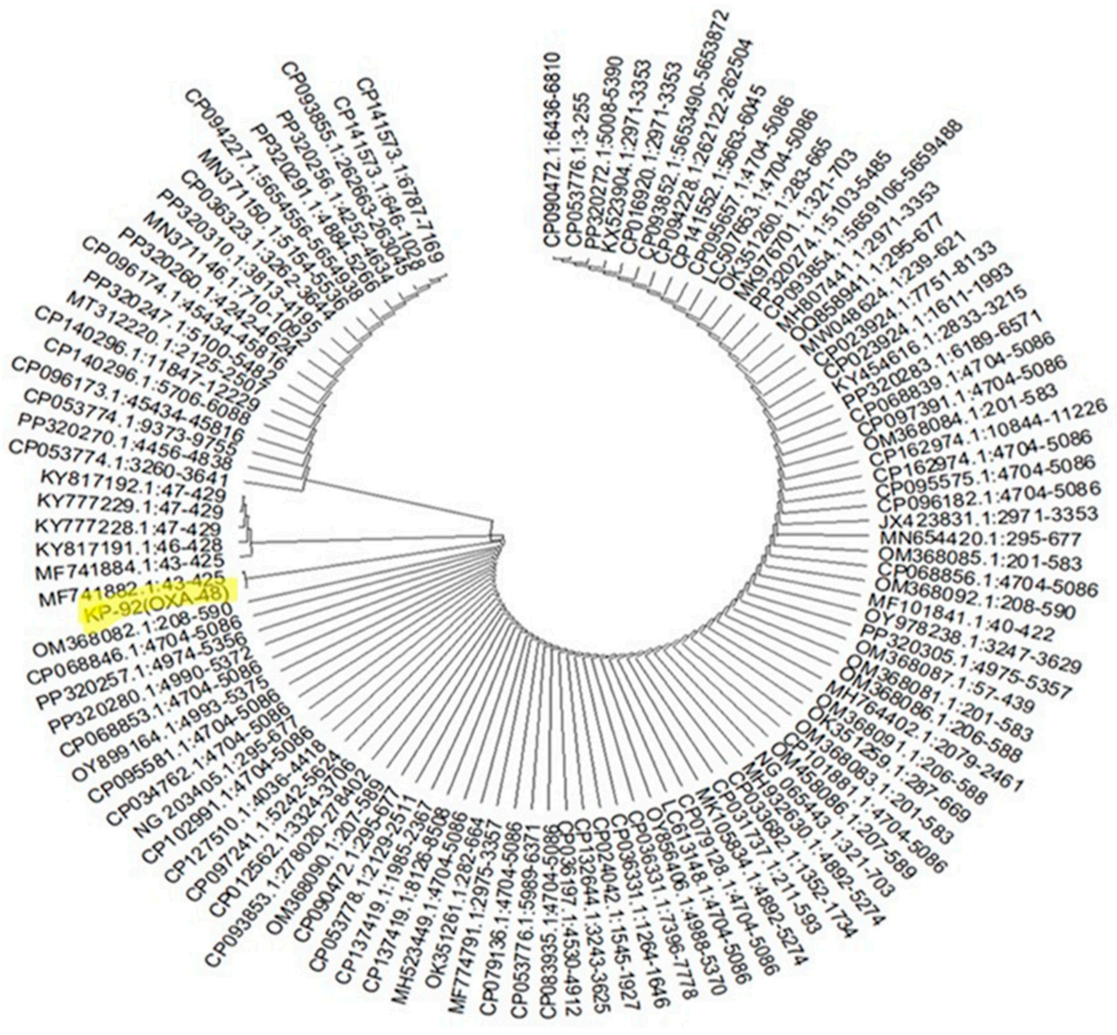

Figure S5. Maximum-likelihood phylogenetic tree of *blaOXA-48*-like sequences from *Klebsiella pneumoniae* isolate KP-92 (PZ234371) and global reference sequences, indicating widespread dissemination.

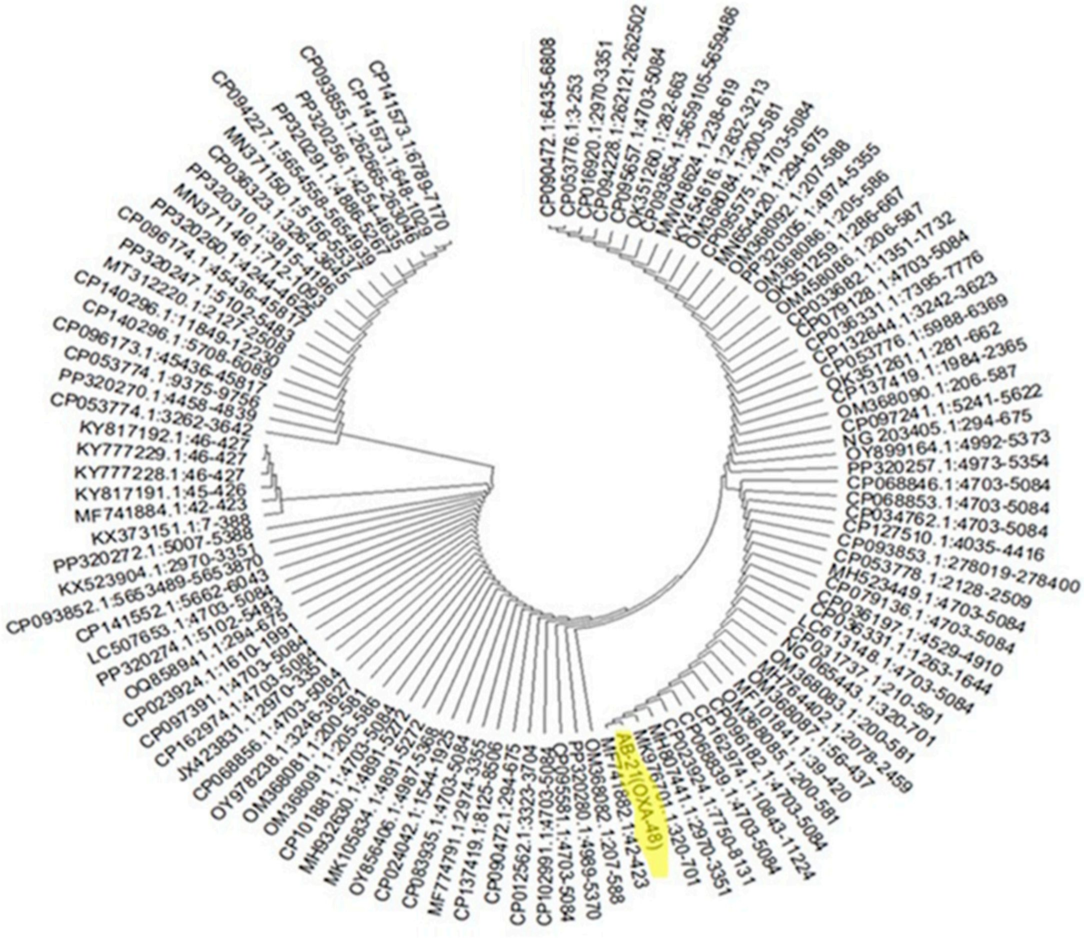

Figure S6. Maximum-likelihood phylogenetic tree (circular dendrogram) of *blaOXA-48* of *A. baumannii* isolate AB-21(PZ201986) vs. world-wide blaOXA-48-producing A. baumannii and Enterobacterales. The tree was constructed by means of aligned blaOXA-48 like sequences with the Tamura-Nei model with 1,000 bootstrap replicates. Bootstrap values70% are displayed on the large branches. Sequences generated in this study are highlighted in yellow.

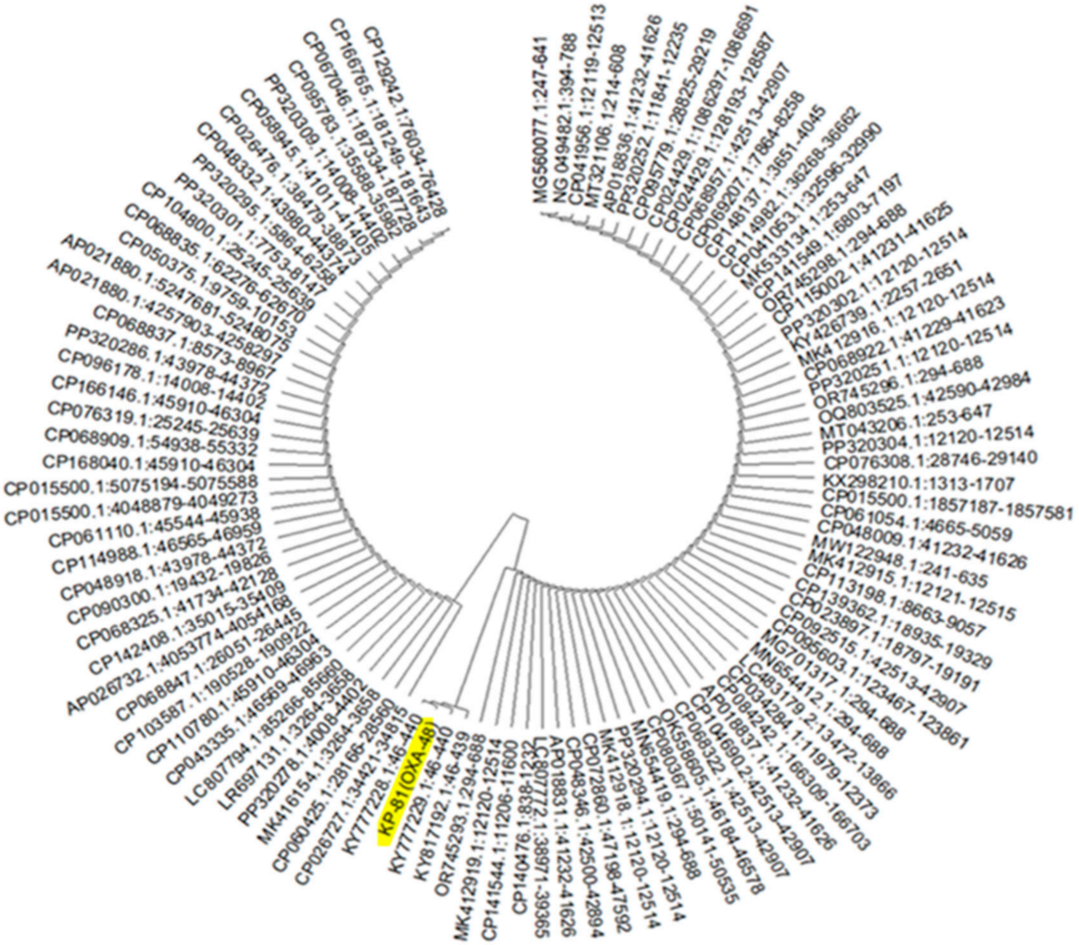

Figure S7. Maximum-likelihood phylogenetic tree of Klebsiella pneumoniae isolate KP-81 (PZ234369) showing clustering with global reference sequences. The tree was constructed using the Tamura–Nei model with 1,000 bootstrap replicates. Sequence-es generated in this study are highlighted in yellow

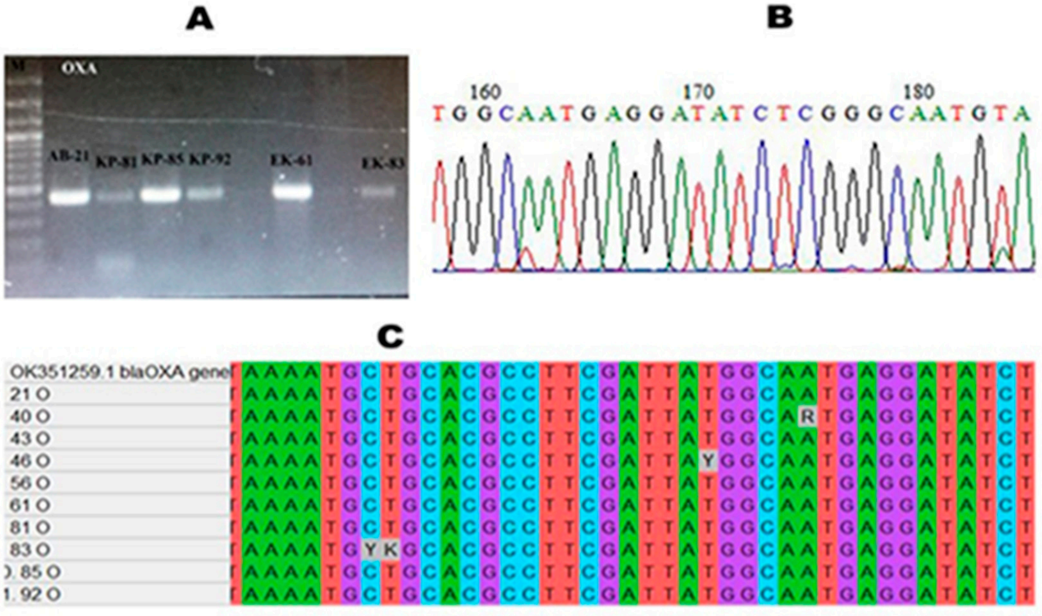

Figure S8. Molecular detection and sequence-based confirmation of the *blaOXA-48*-like gene.

A) Agarose gel electrophoresis of PCR products amplified using *blaOXA-48*-like specific primers. Distinct bands at the expected amplicon size are observed in representative clinical isolates, confirming successful amplification, while the negative control shows no detectable band.

(B) Representative Sanger sequencing chromatogram of the amplified *blaOXA-48* -like gene fragment. Clear, non-overlapping peaks indicate high-quality sequencing, and the displayed nucleotide region corresponds to conserved positions within the blaOXA-48-like gene.

(C) Multiple sequence alignment of the *blaOXA-48*-like gene sequences obtained in this paper with the reference sequence (GenBank accession OK351259.1). Conserved regions are well aligned among the isolates whose difference in nucleotides are merely indicated showing high sequence homology and identification of the gene

Table S1. Primer sequences, target genes, and expected amplicon sizes used for PCR detection of Carbapenemase genes in Gram-negative bacterial isolates

Primer sequences (5′–3′) *(forward and reverse)*	Target gene	Amplicon size (bp)
IMP-F: GGAATAGAGTGGCTT AAYTCTC<br>-R: GGTTTAAYAAAACAACC ACC	*bla*IMP	232
VIM-F: GATGGTGTTTGGTCG CATA<br>R: CGAATGCGCAGCACCAG	*bla*VIM	390
OXA-F: GCGTGGTTAAGGATGAACAC<br>R: CATCAAGTTCAACCCAAC CG	*bla*OXA-48 like	438
NDM-F: GGTTTGGCGATCTG GTTTTC<br>R: CGGAATGGCTCATCACGA TC	*bla*NDM	621
KPC-Fm: CGTCTAGTTCTGCT GTCTTG<br>Rm: CTTGTCATCCTTGTTAGGCG	*bla*KPC	798

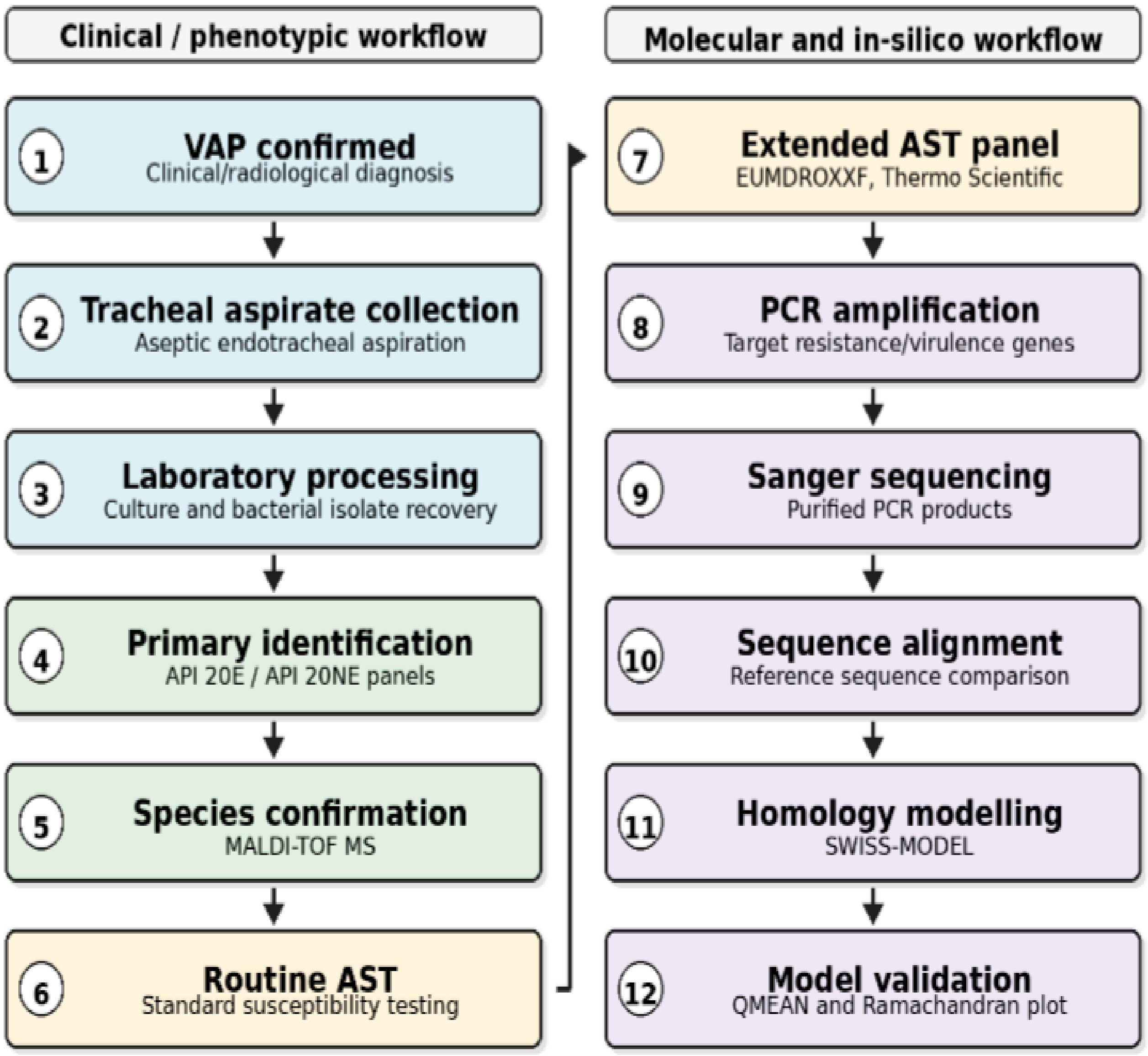

Figure S9. Workflow of phenotypic and molecular characterization of Gram-negative bacilli from VAP tracheal aspirates
